# HIV and Maternal‐Fetal Immunity: Challenges and Strategies for Reducing Vertical Transmission‐A Narrative Review

**DOI:** 10.1002/hsr2.71076

**Published:** 2025-07-15

**Authors:** Emmanuel Ifeanyi Obeagu

**Affiliations:** ^1^ Department of Biomedical and Laboratory Science Africa University Mutare Zimbabwe

**Keywords:** antiretroviral therapy, HIV, maternal‐fetal immunity, placental immunology, vertical transmission

## Abstract

**Background and Aims:**

Mother‐to‐child transmission (MTCT) of HIV remains a significant global health challenge, particularly in resource‐limited settings. The virus disrupts maternal‐fetal immunity through immune dysregulation, placental barrier impairment, and altered antibody transfer, thereby increasing the risk of vertical transmission.

**Methods and Results:**

This narrative review synthesizes current evidence on the immunological and clinical factors influencing MTCT. It highlights how maternal immune exhaustion, placental inflammation, and suboptimal ART timing contribute to transmission. While widespread use of ART has reduced MTCT rates, persistent challenges include late ART initiation, HIV drug resistance, co‐infections, and transmission through breastfeeding. Emerging strategies—such as maternal immune modulation, monoclonal antibody therapies, and tailored ART regimens—show potential for further risk reduction.

**Conclusion:**

To effectively minimize MTCT, comprehensive prevention approaches are essential. These should combine medical interventions with maternal nutrition support, co‐infection management, and safe breastfeeding practices under ART coverage. A multifaceted strategy is key to protecting both maternal and infant health.

## 1 Introduction

Mother‐to‐child transmission (MTCT) of HIV remains a major global health concern, particularly in low‐resource settings with limited access to antiretroviral therapy (ART) and comprehensive maternal care. Without intervention, MTCT risk ranges from 15% to 45%, but with effective ART and preventive strategies, this can be reduced to below 2%, according to the World Health Organization (WHO). Despite this progress, challenges such as delayed ART initiation, poor adherence, and maternal immune dysregulation persist [1, 2, 3, 4, 5]. Maternal‐fetal immunity plays a critical role in determining susceptibility to MTCT. The placenta serves as both a physical and immunological barrier, facilitating nutrient transfer and providing passive immunity through immunoglobulin G (IgG) antibodies. However, HIV disrupts this balance, impairing maternal immune responses and compromising placental integrity. In HIV‐infected pregnant women, immune activation and cytokine dysregulation—particularly elevated levels of TNF‐α and IL‐6—have been associated with increased placental permeability and heightened risk of in utero viral transmission [6, 7, 8, 9, 10].

Moreover, HIV‐induced depletion of CD4 + T cells and functional exhaustion of maternal immune cells reduce the body's capacity to control viral replication. These alterations hinder effective immune defense and contribute to placental dysfunction. HIV also interferes with the expression of placental Fc receptors, leading to reduced transplacental IgG transfer and diminished neonatal immunity. While HIV‐specific neutralizing antibodies have been detected in maternal blood, their role in preventing transmission remains uncertain [4, 11, 12, 13, 14]. Beyond ART, emerging strategies such as monoclonal antibody therapy, cytokine modulation, and placental‐targeted interventions hold promise. Addressing co‐infections and maternal inflammation may further enhance outcomes. This review explores the immunological mechanisms underlying MTCT, identifies key challenges, and evaluates novel strategies to reduce HIV transmission from mother to child [15, 16, 17, 18].

## 2 Aim and Review Methods

This review aims to synthesize current knowledge of maternal‐fetal immunity in HIV‐infected pregnancies, emphasizing immunological mechanisms contributing to vertical transmission. A structured narrative review was conducted using PubMed, Scopus, and Web of Science (2013–2024), with 48 peer‐reviewed articles selected based on relevance to HIV, MTCT, placental immunology, and immune‐based interventions.

## 3 Maternal‐Fetal Immunity in HIV‐Infected Pregnancies

Maternal‐fetal immunity during pregnancy involves a complex balance between immune tolerance and defense, which is disrupted in HIV‐infected pregnancies. Normally, the maternal immune system adapts to tolerate the genetically distinct fetus, preventing rejection through hormonal and immunological changes. However, HIV infection interferes with this balance by targeting crucial immune cells like CD4 + T cells, weakening maternal immunity and increasing viral load during pregnancy. This immune dysregulation leads to chronic inflammation, immune exhaustion, and higher susceptibility to infections and pregnancy complications such as preterm labor and pre‐eclampsia, which can worsen outcomes and elevate the risk of vertical HIV transmission [19, 20, 21, 22]. The fetal immune system is immature and relies largely on maternal immunity for protection. HIV can cross the placenta and expose the fetus to infection at various stages—during pregnancy, labor, or breastfeeding. Although the fetus can produce HIV‐specific antibodies in response to exposure, its limited immune capacity often makes these responses insufficient to prevent infection. The degree of fetal immune response varies with maternal viral load, timing of ART initiation, and gestational age. Early maternal ART significantly lowers transmission risk but does not fully overcome fetal immune limitations [23, 24].

The interaction between maternal and fetal immunity at the placental interface is critical for transmission risk. While the placenta usually acts as a selective barrier, HIV infection can compromise its integrity, especially in the presence of high maternal viral loads and placental inflammation, facilitating viral passage to the fetus. Co‐infections and maternal immune activation further increase transmission risk. Effective ART reduces maternal viral load and is the most successful intervention to prevent vertical transmission. However, incomplete adherence, late ART initiation, or resistant HIV strains can still result in fetal infection [21, 25, 26].

## 4 Immune Modulation and ART in HIV‐Infected Pregnancies

ART is central to reducing vertical HIV transmission by suppressing maternal viral load and modulating immune activation. ART lowers systemic inflammation, restores partial immune function, and reduces placental exposure to HIV. This immune stabilization protects both mother and fetus, decreasing the risk of in utero infection. Early initiation and strict adherence to ART are critical. While ART does not eliminate fetal exposure, it significantly minimizes transmission risk. Complementary interventions—such as cesarean delivery in cases of high viral load and infant postnatal prophylaxis—further reduce transmission. ART regimens must be individualized based on maternal health and resistance profiles [19, 27, 28].

## 5 Challenges in Preventing Vertical Transmission in Pregnancy

Despite advances in ART and preventive strategies, preventing MTCT of HIV during pregnancy remains complex—particularly in resource‐limited settings. Challenges span medical, socioeconomic, and systemic domains that limit the effectiveness of current interventions [29].

### 5.1 Inconsistent Access to ART

In many low‐ and middle‐income countries, access to ART is uneven due to weak healthcare infrastructure, frequent stockouts, high medication costs, and a shortage of trained providers. These factors lead to treatment interruptions, increasing maternal viral load and the risk of MTCT during pregnancy, delivery, or breastfeeding [30].

### 5.2 Nonadherence to ART Regimens

Even where ART is available, non‐adherence significantly threatens prevention efforts. Pregnant women may struggle with medication side effects, pill burden, stigma, or lack of understanding about ART's importance. Pregnancy‐related symptoms, such as nausea or fatigue, can worsen adherence, and limited access to regular monitoring impedes the timely detection of treatment failure [31].

### 5.3 Late HIV Diagnosis in Pregnancy

Late or missed HIV diagnosis remains a critical barrier. In many regions, HIV screening is not routinely integrated into antenatal care, resulting in some women learning of their status only late in pregnancy or even during labor. Delayed diagnosis restricts the window for initiating ART and achieving viral suppression, undermining MTCT prevention efforts [32].

### 5.4 Stigma and Discrimination

Stigma related to HIV continues to deter women from seeking testing, disclosing their status, or adhering to treatment. Fear of judgment or rejection may lead to isolation, disengagement from care, or discontinuation of ART. This social barrier undermines both individual health and public health efforts to reduce transmission [33].

### 5.5 Limited Access to Comprehensive Maternal and Child Health Services

ART alone is insufficient without robust maternal and child health services, including antenatal care, skilled delivery support, and postnatal monitoring. In underserved regions, women may lack access to regular viral load testing, HIV counseling, or safe delivery environments—factors that increase peripartum transmission risk. Gaps in postpartum care also hinder timely initiation of infant prophylaxis [34].

### 5.6 Socioeconomic and Structural Barriers

Poverty, limited education, food insecurity, and poor transportation infrastructure restrict access to consistent healthcare. Women facing economic hardship may prioritize immediate survival needs over long‐term health, increasing the likelihood of late presentation, nonadherence, and poorer outcomes. Malnutrition and other social determinants further compromise maternal immunity and ART effectiveness [35].

### 5.7 Inadequate Postpartum HIV Care and Infant Prophylaxis

Postnatal care is often underemphasized in MTCT programs. HIV‐exposed infants require continued monitoring and prophylaxis, especially when maternal viral suppression was suboptimal. However, many women discontinue ART postpartum due to a lack of support or misconceptions. This gap in care increases the risk of HIV transmission during breastfeeding and impairs early infant diagnosis and treatment [20].

### 5.8 Biological and Immunological Factors

Pregnancy induces changes in the maternal immune system, affecting the maternal‐fetal interface. Immune activation, chronic inflammation, and co‐infections such as STIs or bacterial vaginosis may increase placental permeability and facilitate HIV transmission. Furthermore, individual immunogenetic variations can affect susceptibility. Addressing these biological complexities requires ongoing research to develop targeted immune‐based interventions [20].

## 6 Strategies to Reduce Vertical Transmission of HIV in Pregnancy

Reducing vertical transmission of HIV during pregnancy requires a multifaceted approach. Early HIV testing and diagnosis, ideally in the first trimester, enable timely initiation of ART, which significantly lowers maternal viral load and transmission risk. Routine testing should be repeated in late pregnancy for high‐risk women. Ensuring ART adherence, managing co‐infections, promoting safe delivery practices—such as elective cesarean section when viral load exceeds 1000 copies/mL—and supporting exclusive breastfeeding under ART coverage are essential strategies. Comprehensive prenatal care, including nutritional support and psychosocial counseling, further enhances maternal‐fetal health and reduces the risk of MTCT [36, 37].

## 7 WHO Recommendations on ART in Pregnancy

The WHO emphasizes early initiation of ART during pregnancy to prevent MTCT of HIV. ART should begin as soon as HIV is diagnosed, regardless of gestational age or CD4 count, to achieve rapid maternal viral suppression and reduce in utero and intrapartum transmission risks. Early treatment also improves maternal immune recovery and perinatal outcomes [19]. WHO recommends a once‐daily fixed‐dose combination of Tenofovir disoproxil fumarate (TDF, 300 mg), Lamivudine (3TC, 300 mg), and Dolutegravir (DTG, 50 mg) as the first‐line regimen in pregnancy. This combination is favored for its high efficacy, safety profile, and rapid viral load reduction. Dolutegravir, in particular, shows faster viral suppression than earlier regimens. Although initial concerns existed about neural tube defects with DTG, newer data indicate the risk is minimal, leading WHO to recommend its continued use during pregnancy and breastfeeding [29]. Standard dosing remains unchanged throughout pregnancy and postpartum, as pharmacokinetic studies show no need for adjustment. However, routine monitoring of maternal renal and liver function, along with viral load, is essential to ensure safety and effectiveness. WHO's guidance supports integrating ART with broader maternal health services to optimize outcomes for both mother and child.

## 8 Elective Cesarean Section (C‐Section)

For HIV‐positive pregnant women with a maternal HIV RNA viral load > 1000 copies/mL near term, an elective cesarean section (C‐section) before the onset of labor is generally recommended to reduce the risk of vertical transmission. Vaginal delivery in the context of a high maternal viral load increases the infant's exposure to HIV‐infected blood and genital secretions, raising transmission risk. Elective C‐section is particularly important for women with late HIV diagnosis, incomplete ART response, or unsuppressed viral load. Current WHO and CDC guidelines define viral suppression as < 50 copies/mL, under which vaginal delivery is considered safe and preferred. The decision for C‐section should be individualized, carefully balancing maternal and fetal risks and benefits [38, 39, 40].

## 9 Breastfeeding Practices

Breast milk can transmit HIV; thus, avoidance is advised where safe and affordable alternatives exist. In resource‐rich settings, exclusive formula feeding is recommended. However, in resource‐limited environments where formula may be unsafe or unaffordable, exclusive breastfeeding combined with strict maternal ART adherence and ongoing viral suppression remains the preferred strategy. Regular viral load monitoring during breastfeeding is essential to minimize transmission risk [41].

## 10 Infant Antiretroviral Prophylaxis and Testing

Newborns should receive antiretroviral prophylaxis within hours of birth, typically for 4–6 weeks, regardless of maternal ART efficacy. Early infant diagnosis using PCR testing at birth and during infancy ensures timely detection and treatment if needed [42].

## 11 Managing Pregnancy Complications and Co‐Infections

Conditions like preterm labor, intrauterine growth restriction (IUGR), and co‐infections (e.g., syphilis, tuberculosis, STIs) heighten transmission risk by compromising placental integrity and increasing inflammation. Routine screening, treatment, and maternal nutritional support are crucial components of care [43].

## 12 Comprehensive Care and Psychosocial Support

Optimal outcomes require skilled birth attendance, continued maternal ART postpartum, infant follow‐up, and psychosocial counseling to address stigma, mental health, and adherence challenges. Integrated maternal‐child health services, coordinated among obstetricians, HIV specialists, pediatricians, and community workers, are vital for reducing vertical transmission and advancing global elimination goals [4].

## 13 Prevention Strategies for Reducing Vertical Transmission of HIV

Preventing vertical transmission of HIV requires a comprehensive approach integrating maternal ART, neonatal prophylaxis, EID, and strategic delivery planning. Each intervention is essential in disrupting maternal‐fetal HIV transmission and is supported by immunological principles and clinical guidelines [37].

### 13.1 Maternal ART Regimens

Maternal ART is the cornerstone of prevention. The WHO recommends initiating ART as early as possible in pregnancy, regardless of CD4 count, to achieve rapid viral suppression before delivery [38].

- **Preferred regimen**: A fixed‐dose combination of tenofovir disoproxil fumarate (TDF), lamivudine (3TC) or emtricitabine (FTC), and dolutegravir (DTG) is the first‐line option. DTG is favored for its rapid viral suppression, high genetic barrier to resistance, and strong maternal‐fetal safety data.
- **Alternative regimens**: In settings where DTG is unavailable or contraindicated (e.g., early pregnancy concerns), alternatives include efavirenz (EFV) or lopinavir/ritonavir (LPV/r) with TDF and 3TC/FTC [39].

Early initiation of ART—ideally pre‐conception or during the first trimester—limits immune activation and preserves placental integrity, reducing inflammatory mediators that increase placental permeability to HIV.

### 13.2 Neonatal Prophylaxis

Postnatal antiretroviral prophylaxis further reduces transmission risk.

- **Standard prophylaxis**: For low‐risk infants (mothers with viral suppression), daily nevirapine for 4–6 weeks is recommended.
- **Enhanced prophylaxis**: For high‐risk infants (e.g., maternal viremia, late ART), dual prophylaxis with nevirapine and zidovudine is given for 6–12 weeks to provide broader protection during early life [40].

This dual approach addresses different stages of viral replication and supports the immature neonatal immune system.

### 13.3 Early Infant Diagnosis

Early detection of infant HIV is critical for timely treatment. Maternal antibodies can persist for up to 18 months, making serology unreliable. Therefore, nucleic acid amplification tests (NAATs), such as PCR, are used [41].

- **Testing protocol**: WHO recommends dried blood spot (DBS) PCR testing at 6 weeks of age. A positive result warrants confirmatory testing and immediate ART initiation. If negative, further testing at 9 months and after breastfeeding cessation is essential to detect postnatal transmission.

Expanding access to point‐of‐care testing platforms allows for faster diagnosis and linkage to care, especially in resource‐limited settings [42].

### 13.4 Cesarean Section

Cesarean section reduces peripartum HIV transmission when maternal viral suppression is not achieved.

- **Timing**: Elective C‐section is recommended at 38 weeks, before labor or membrane rupture, to minimize exposure to maternal blood and secretions.
- **Immunological basis**: C‐section reduces mechanical trauma and mucosal contact during delivery, limiting pathways for viral entry [43].
- **Considerations**:
  ◦ *Pros:* Reduced transmission risk in women with high viral loads or poor ART adherence.
  ◦ *Cons:* Surgical risks, longer recovery, and future pregnancy complications; may be less feasible in low‐resource settings [4].

In women with effective ART and undetectable viral load, vaginal delivery is generally safe and preferred.

## 14 Future Directions in Reducing Vertical Transmission of HIV During Pregnancy

Future strategies to reduce vertical HIV transmission must focus on innovation, equity, and sustainability, particularly in resource‐limited settings. Early diagnosis remains critical; expanding point‐of‐care and self‐testing can improve timely ART initiation, especially in underserved areas. Decentralizing ART services through community health posts and mobile clinics can enhance access [19, 29]. Enhancing maternal immune health through integrated antenatal care—including nutritional support, immunization, and management of co‐infections like tuberculosis and malaria—may reduce inflammation at the maternal‐fetal interface, lowering transmission risk. Personalized ART regimens, informed by pharmacogenomic testing and long‐term safety studies of drugs like dolutegravir, are needed to optimize treatment and minimize fetal toxicity. Broader access to viral load monitoring will help detect treatment failure early and allow timely regimen adjustments [30, 31].

For infants, simplified prophylaxis protocols and long‐acting antiretroviral formulations could improve adherence and reduce caregiver burden. Expanding point‐of‐care early infant diagnosis platforms is essential for prompt detection and treatment initiation. Strengthening health systems through training, task‐shifting, reliable drug supplies, and digital tools can improve care continuity and maternal‐infant tracking [32, 33]. Addressing social determinants is crucial: community education, male partner involvement, and integration of HIV services with general maternal‐child health programs can reduce stigma and improve uptake. Future programs must prioritize equitable access by overcoming transportation, cultural, and gender barriers, while funding should support local manufacturing and subsidized care to sustain progress in vulnerable populations [34].

## 15 Limitations

This review faced limitations including study heterogeneity, varied designs, populations, and treatment protocols, limiting generalizability. Gaps remain in understanding maternal‐fetal immune interactions and long‐term fetal effects of HIV exposure. A lack of large, multicenter studies on maternal‐fetal immunity in diverse HIV‐affected populations also limits current knowledge.

## 16 Conclusion

Significant progress has been made in preventing vertical HIV transmission, yet challenges remain. Effective management requires a multifaceted approach combining advanced ART, personalized care, and integrated health services. Future efforts must focus on innovations like long‐acting ART, improved testing, maternal vaccines, and enhanced infant prophylaxis. Addressing social barriers such as stigma and healthcare disparities is also crucial. Ensuring equitable access, especially in resource‐limited settings, is essential to eliminate MTCT and improve outcomes for mothers and children worldwide.

## Author Contributions

**Emmanuel Ifeanyi Obeagu:** conceptualization, writing – original draft, writing – review and editing, visualization, validation, methodology, supervision.

## Conflicts of Interest

The author declares no conflicts of interest.

## Transparency Statement

The lead author Emmanuel Ifeanyi Obeagu affirms that this manuscript is an honest, accurate, and transparent account of the study being reported; that no important aspects of the study have been omitted; and that any discrepancies from the study as planned (and, if relevant, registered) have been explained.

## Data Availability

Data sharing is not applicable to this article as no new data were created or analyzed in this study.
